# Thermal remote sensing reveals communication between volcanoes of the Klyuchevskoy Volcanic Group

**DOI:** 10.1038/s41598-021-92542-z

**Published:** 2021-06-22

**Authors:** Diego Coppola, Marco Laiolo, Francesco Massimetti, Sebastian Hainzl, Alina V. Shevchenko, René Mania, Nikolai M. Shapiro, Thomas R. Walter

**Affiliations:** 1 Dipartimento Di Scienze Della Terra, Università Di Torino, Turin, Italy; 2 Centro Interdipartimentale Sui Rischi Naturali in Ambiente Montano E Collinare, Università Di Torino, Turin, Italy; 3 GFZ German Research Centre for Geosciences, Telegrafenberg, 14473 Potsdam, Germany; 4 Institute of Volcanology and Seismology FEB RAS, Piip boulevard 9, Petropavlovsk-Kamchatsky, 683006 Russia; 5 Institut de Sciences de La Terre, CNRS (UMR5275), Université Grenoble Alpes, Grenoble, France; 6 Schmidt Institute of Physics of the Earth, Russian Academy of Sciences, Moscow, Russia

**Keywords:** Volcanology, Natural hazards

## Abstract

Volcanoes are traditionally considered isolated with an activity that is mostly independent of the surrounding, with few eruptions only (< 2%) associated with a tectonic earthquake trigger. Evidence is now increasing that volcanoes forming clusters of eruptive centers may simultaneously erupt, show unrest, or even shut-down activity. Using infrared satellite data, we detail 20 years of eruptive activity (2000–2020) at Klyuchevskoy, Bezymianny, and Tolbachik, the three active volcanoes of the Klyuchevskoy Volcanic Group (KVG), Kamchatka. We show that the neighboring volcanoes exhibit multiple and reciprocal interactions on different timescales that unravel the magmatic system’s complexity below the KVG. Klyuchevskoy and Bezymianny volcanoes show correlated activity with time-predictable and quasiperiodic behaviors, respectively. This is consistent with magma accumulation and discharge dynamics at both volcanoes, typical of steady-state volcanism. However, Tolbachik volcano can interrupt this steady-state regime and modify the magma output rate of its neighbors for several years. We suggest that below the KVG the transfer of magma at crustal level is modulated by the presence of three distinct but hydraulically connected plumbing systems. Similar complex interactions may occur at other volcanic groups and must be considered to evaluate the hazard of grouped volcanoes.

## Introduction

Closely located or clustered volcanoes may become conjointly active and are hence considered especially hazardous, yet robust evidence for their connectivity remains sparse. Examples of such a synchronized volcanic activity are discussed for neighboring volcanoes in Iceland^1^, Alaska^2^, Kamchatka^3,4^, Italy^5^, and elsewhere^6,7^, although larger time-scale synchronicity has been also reported for global volcanism^8^. Reasons for the linked activity of adjacent volcanoes are only poorly understood and may be locally different, including triggering by large tectonic earthquakes and associated stress changes within the crust^9–14^, and the competition of volcanoes for common reservoirs^3,15^. Conjoint unrest and deformation activity at clustered volcanoes occurs with temporal delays of days to months (or even more) and appears to be distance-dependent^7^. Magmatic sources spaced less than about 10 km apart tend to interact, whereas those spaced over 25 km do not^7^. However, interactions over longer distances (>20 km) have been hypothesized for volcanoes that share a common deep source, or in response to large dike intrusions or subduction earthquakes^7^. While observations suggest positively correlated feedback (one volcano triggers the other one), only a few examples^5,16^ underline the existence of anti-correlated activity (i.e., volcanic unrest may shut down activity in the neighborhood).

Previous studies of correlated and anti-correlated volcanism are mainly based on poor data and reduced or biased eyewitness accounts. This is because reports often describe a singular, possibly sporadic occurrence of conjoint activity change, where eruptions are more likely to be reported than periods of quiescence and decreasing activity. Robust and statistically significant testing of repeat observations was not yet achieved and may overcome some of the reporting limitations.

Here we employed a unique time-series of satellite thermal data (from 2000 to 2020) derived from the Moderate Resolution Imaging Spectroradiometer (MODIS) sensor^17^, to study potential volcano interactions within the Klyuchevskoy Volcano Group (KVG) in Kamchatka (Russia). The KVG hosts three adjacent (~ 10 to 30 km distant) active volcanoes (Klyuchevskoy, Bezymianny, and Tolbachik), with contrasting eruptive products that possibly indicate different magmatic sources and a compound multilevel feeding system^18,19^. Whether this composite feeding system forms a single interconnected trans-crustal magmatic system^20^, and in which degree the three volcanoes interact remains so far an open question^21,22^.

The Klyuchevskoy Volcanic Group (KVG) has been monitored for > 50 years by the Kamchatkan Branch of the Geophysical Survey of the Russian Academy of Sciences (KBGS). Due to harsh climate conditions, the monitoring system mainly consists of seismic stations and webcams. Nowadays, 17 telemetric stations transmit the data to the central monitoring office, where it is being analyzed in real-time^23^ and allowed to investigate the deep structure of KVG^24^. Although localized and dense seismic networks were installed for short-term times^25,26^, these experimental networks do not permit decade-long analysis and robust statistics, so that details on eruption occurrence and eruption rates remained hidden. As this work shows, satellite thermal remote sensing reveals complex interactions under the KVG, whereby each volcano is able to influence its neighbors.

## The Klyuchevskoy Volcanic Group (KVG)

**Figure 1.**
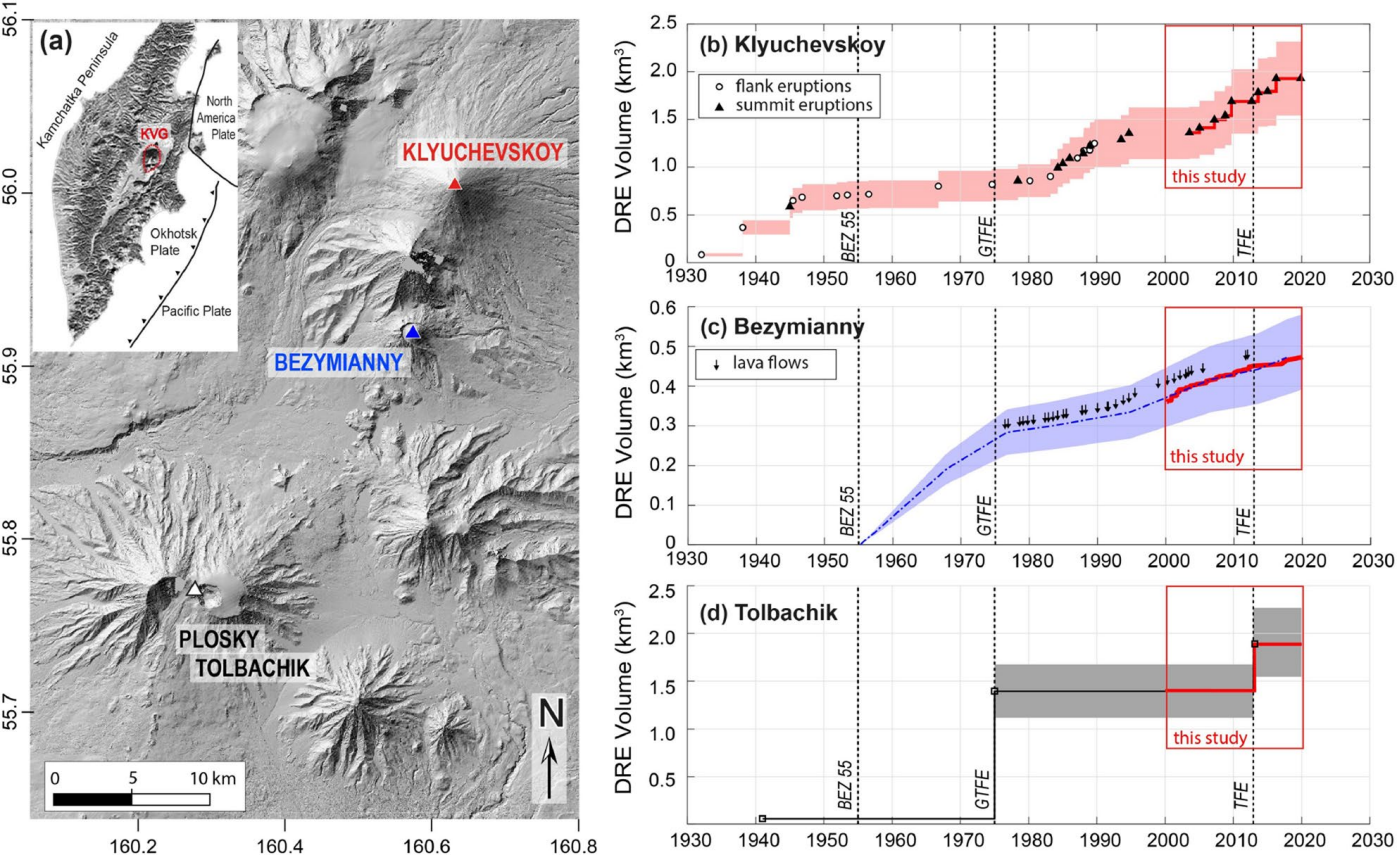
(**a**) Distribution of volcanic centers within the Klyuchevskoy Volcanic Group (KVG). Right panels show the Cumulative Dense Rock Equivalent (DRE) volumes of lavas erupted between 1930 and 2020 for (**b**) Klyuchevskoy^21^, (**c**) Bezymianny^27^, and (**d**) Tolbachik^28^. All volumes are recalculated for DRE using: (i) a magma density of 2800 kg m^−3^ and an average density of lava flows of 2500 kg m^−3^ for Klyuchevskoy and Tolbachik^28^; (ii) a magma density of 2500 kg m^−3^ and an average density of lava dome of 2000 kg m^−3^ for Bezymianny^29^. A standard error of ± 20% (colored fields) takes into account uncertainties in the estimates of bulk volumes and densities of erupted products. Red bold lines within insets, correspond to satellite-derived volumes (this work; see “Methods”). The grey bars outline the timing of the Bezymianny eruption in 1955–56 (BEZ55), the Great Tolbachik Fissure Eruption in 1975 (GTFE) and the Tolbachik Fissure Eruption in 2012 (TFE). Shaded relief map derived from ArcticDEM digital elevation model (https://www.pgc.umn.edu/data/arcticdem/) and elaborated using QGIS version 3.16.3 (http://qgis.osgeo.org); Time series generated using MATLAB software (www.mathworks.com).

The KVG is a prominent volcanic massif located in the northern part of the Central Kamchatka Depression. Dozens of volcanic centers were built during the construction of the massif, which currently has three active volcanoes^21^: Klyuchevskoy, Bezymianny, and Tolbachik (Fig. 1a). The KVG is a very active and relatively young volcanic group mainly developed during the last 300–400 ka^30^. Volcanism is fed by sub-arc mantle, melted under an influx of melts and fluids from the subducting Pacific plate^31–35^. Additional influx of hot mantle following recent slab detachment^36^, and interaction with metasomatized mantle^37,38^ contribute to the exceptional level of volcanic activity in the area and the very diverse volcanic manifestations and products. Seismic activity of the KVG volcanoes is abundant and includes long periods of sustained tremors as well as numerous volcano-tectonic (VT) and long-period (LP) events. The latter mostly occur at two depth ranges: above 5 km and close to 30 km^22^. Geophysical and petrologic data have been used to infer that all the KVG volcanoes are fed by a common parental magma^21,39^. However, different isotope compositions of rocks from Klyuchevskoy and Bezymianny^19,37^ do not support such view, in favor of multiple magma sources, with only limited interaction.

Klyuchevskoy volcano (4750 m.a.s.l) is the highest in the group and one of the world’s most active volcanoes. Its recent activity is characterized by the effusion of voluminous basaltic andesite lava flows, often associated with moderate to violent explosive activity. Between 1930 and 2005, the volcano has erupted an estimated ~ 1.5 × 10^9^ m^3^ of lava^40^ (dense rock equivalent; DRE), with an average magma output rate (DRE) of ~ 0.67 m^3^ s^−1^. The output rate accelerated since 1978, associated with a change in the eruptive pattern that shifted from flank- to summit-dominated eruptions^40^ (Fig. 1b). After nine years of rest, in 2003, the volcano began a new activity phase characterized by nine summit eruptions until December 2019^41^. Seismic, geodetic, and petrographic data^18,21,22,25,40^ suggest that Klyuchevskoy’s eruptions are fed through a sub-vertical, pipe-like conduit extending to a depth of 30–50 km below the volcano, where the primary magma reservoir is located. On its way to the surface, the magma is stored at a depth of 15–25 km and then transported further upwards to a shallow (3–5 km deep) peripheral reservoir^24^. During ascent, the magma evolves from high-Mg low-Al basalt to low-Mg high-Al basaltic andesite^42^, making it different from the eruptive products of the other active KVG volcanoes^39^. Eventually, before the conduit reaches the summit crater, numerous radial dikes depart and feed eruptions at the mid- and lower-volcano flanks^21,40^.

Bezymianny is an andesitic volcano (2882 m.a.s.l.) that reawakened in 1955–56 with a paroxysmal eruption (VEI 5) that disrupted the old cone forming a large horseshoe-shaped crater^43^. Since then, near-continuous lava dome growth was accompanied by mostly explosive activity^29,44^. The greatest rate of dome growth occurred during the first two decades until 1977 (Fig. 1c), when lava flows were observed for the first time marking a pivotal change in the volcano’s dome growth mechanism^27,44,45^. Ever since then, Bezymianny’s eruptions showed a recurrent cyclical behavior depicted by extrusive-explosive-effusive activity^29,44^. Previous works^46–50^ outlined how this cyclic activity was accompanied by precursory thermal radiation preceding the explosive events by few weeks to days. Until 2017, more than 55 distinct episodes of dome-growth filled most of the 1956 collapse amphitheater^41,44^ gradually developing a stratocone with an average growth rate^27^ of ~ 0.30 m^3^ s^−1^ (blue line in Fig. 1c). Geophysical and petrological data suggest a multi-level magma plumbing system beneath Bezymianny volcano with at least three crustal reservoirs located between 10 and 18 km, 5–8 km, and < 2 km depth^18,26,51–55^.

The Tolbachik massif comprises two large stratocones, Ostry (“Sharp”) Tolbachik (3682 m.a.s.l.) and Plosky (“Flat”) Tolbachik (3085 m.a.s.l.), in the southernmost part of the KVG^56^ at approximately 30 km distance to Kluchevskoy and ~ 20 km distance to Bezymianny (Fig. 1a). A 70 km long zone of monogenetic basaltic cones extends across the Plosky Tolbachik cone; whose southern branch was the place of the 1975–1976 Great Tolbachik Fissure Eruption (GTFE)^57^. This eruption produced extensive lava fields composed of high-magnesium and high-aluminum basalts, from northern and southern vents, respectively^56^. With a total DRE volume of ~ 1.5 × 10^9^ m^3^ (Fig. 1d), it was one of the largest basaltic eruptions in Kamchatka during historical times^57^. After the GTFE, no signs of activity were recorded until November 2012, when increased seismic activity heralded the beginning of a new eruption^28^. The 2012–2013 eruption took place at the south flank of the Plosky Tolbachik cone and was dominated by Hawaiian-style activity associated with an emplacement of a large lava field^58^. During the 205 days of activity, a lava volume of ~ 0.5 × 10^9^ m^3^ was erupted, with a gently declining trend throughout the whole eruptive period^58^. Satellite geodesy could reveal the intrusion of a 6.1 km long dike intrusion, opening up to 8 m, adding almost 10% to the total eruption volume^59^.The activity ceased entirely by the end of August 2013. According to Koulakov et al.^18^, one magmatic pathway of Tolbachik appears to be connected with the marginal part of the Klyuchevskoy deep reservoir, and another seems to originate from an independent mantle source located to the south of Tolbachik.

## Remote sensing of eruption effusion rates

**Figure 2:**
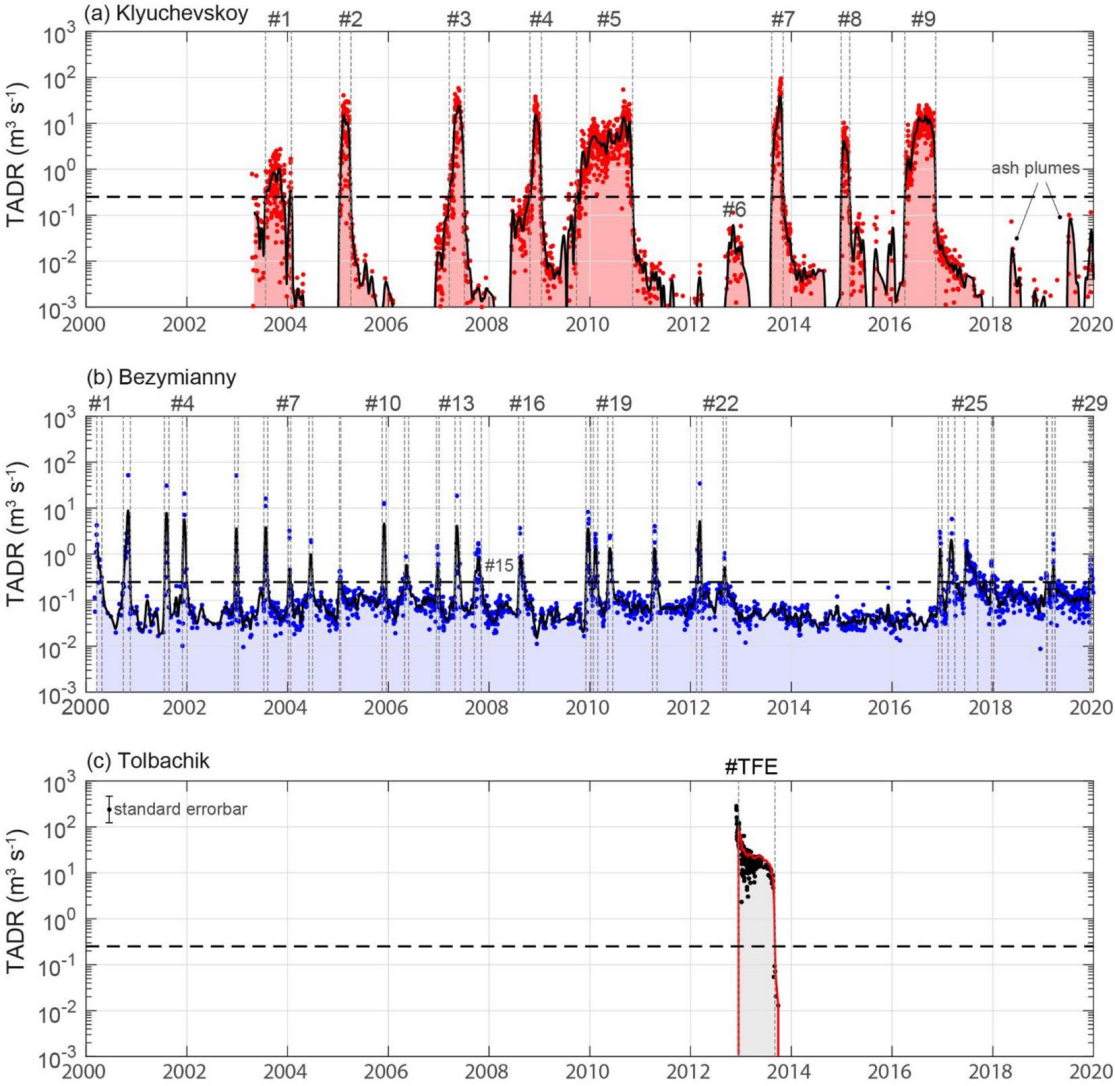
Time-series of the time-averaged lava discharge rate (TADR; logarithmic scale) for (**a**) Klyuchevskoy, (**b**) Bezymianny, and (**c**) Tolbachik. Single satellite measurements (dots) are interpolated and smoothed (line) to provide continuous data. The horizontal dashed lines represent the threshold of 0.25 m^3^ s^−1^ used for automatized detection of eruptions (see “Methods” section). The start and end of each eruptive period (labelled on the top of each plot) are marked by the vertical dashed lines. The error for every single datapoint is ± 50%. The time series is generated using MATLAB software (www.mathworks.com).

We calculated the time-averaged lava discharge rate (TADR) and the erupted lava volumes at the three volcanoes by using MODIS infrared data acquired between March 2000 and December 2019 (bold lines in insets of Fig. 1b–d) determined with the MIROVA system^17,60^. Details of the methodology and associated limits are described in the “Methods” section accompanying this paper. A TADR threshold of 0.25 m^3^ s^−1^ is used to automatically recognize the main eruptive periods at each volcano (Fig. 2), and to quantify the eruption parameters summarized in Tables 1 and 2.

**Table 1:** Eruptions and parameters of Klyuchevskoy volcano retrieved from MODIS data.

Eruption #	Start (dd/mm/yyyy hh:mm)	Peak (dd/mm/yyyy hh:mm)	End (dd/mm/yyyy hh:mm)	Duration (days)	dt_es (days)	Vol (× 10^6^ m^3^)	TADRmax (m^3^ s^−1^)	MOR (m^3^ s^−1^)
1	23/07/2003 16:10	07/11/2003 10:10	24/01/2004 16:05	186.90	N.d	7.3	2.7	0.5
2	14/01/2005 15:40	07/02/2005 16:25	28/03/2005 11:30	73.60	356.0	52.3	40.7	8.2
3	18/03/2007 15:35	22/05/2007 10:35	18/06/2007 10:15	103.50	720.2	85.4	58.2	9.5
4	15/10/2008 16:20	30/11/2008 10:50	07/01/2009 15:55	86.20	485.3	45.2	38.2	6.1
5	20/09/2009 10:10	28/08/2010 11:10	28/10/2010 16:25	441.60	255.8	151.5	54.1	4.0
6*	09/09/2012 15:35	04/11/2012 10:40	07/01/2013 10:40	119.80	681.97	0.15	0.1	0.01
7	13/08/2013 15:10	16/10/2013 11:05	25/10/2013 11:00	75.10	218.2	94.6	93.9	14.6
8	01/01/2015 10:05	14/01/2015 15:15	23/02/2015 10:25	55.80	433.0	12.3	10.2	2.6
9	06/04/2016 15:20	05/09/2016 14:25	01/11/2016 16:00	211.80	408.2	137.8	24.8	7.5
10**	11/11/2019	n.d	n.d	n.d	1104.333	n.d	n.d	n.d

Volumes, TADR and MOR are calculated as bulk values (see “Methods”).

*Eruption #6 was detected by MODIS but with TADR < 0.25 m^3^ s^−1^. Eruption start and end, are selected manually based on first and last thermal anomalies.

**Start of Eruption #10 from^39^.

**Table 2:** Eruptions and parameters of Bezymianny retrieved from MODIS data.

Event #	Date Exp.*	Start	Peak	End	Duration (days)	dt_pp (days)	dt_es (days)	Vol (× 10^6^ m^3^)	TADRmax (m^3^ s^−1^)	MOR (m^3^ s^−1^)
1	13/03/2000	04/03/2000 05:30	18/03/2000 11:35	24/04/2000 08:01	51.1	nan	nan	3.5	4.2	0.8
2	01/11/2000	27/09/2000 15:42	01/11/2000 11:05	16/11/2000 18:10	50.1	228.0	156.3	14.2	51.9	3.3
3	06/08/2001	22/07/2001 09:52	06/08/2001 10:25	23/08/2001 21:20	32.5	278.0	247.7	10.9	30.9	3.9
4	16/12/2001	28/11/2001 17:20	13/12/2001 11:05	01/01/2002 23:58	34.3	129.0	96.8	8.1	20.7	2.7
5	25/12/2002	12/12/2002 20:43	25/12/2002 14:55	07/01/2003 20:17	26.0	377.2	344.9	4.8	50.9	2.1
6	26/07/2003	13/07/2003 00:25	26/07/2003 15:15	10/08/2003 16:45	28.7	213.0	186.2	5.3	16.1	2.1
7	13/01/2004	06/01/2004 17:04	14/01/2004 10:00	23/01/2004 22:43	17.2	171.8	149.0	0.6	3.2	0.4
8	18/06/2004	03/06/2004 22:01	19/06/2004 15:10	30/06/2004 13:43	26.7	157.2	132.0	1.6	2.0	0.7
9	11/01/2005	10/01/2005 13:02	16/01/2005 15:40	20/01/2005 22:28	10.4	211.0	194.0	0.3	0.4	0.3
10	30/11/2005	16/11/2005 15:08	30/11/2005 11:50	16/12/2005 00:15	29.4	317.8	299.7	6.4	12.8	2.5
11	09/05/2006	29/04/2006 07:39	08/05/2006 11:05	27/05/2006 23:59	28.7	159.0	134.3	1.1	0.9	0.5
12	24/12/2006	15/12/2006 18:15	23/12/2006 11:25	03/01/2007 10:45	18.7	229.0	201.8	0.8	1.5	0.5
13	11/05/2007	29/04/2007 13:39	12/05/2007 15:55	07/06/2007 08:13	38.8	140.2	116.1	6.1	18.5	1.8
14	14/10/2007	16/09/2007 08:57	15/10/2007 14:40	05/11/2007 22:31	50.6	155.9	101.0	2.3	1.7	0.5
15*	05/11/2007	N.d	05/11/2007	N.d	N.d	N.d	N.d	N.d	N.d	N.d
16	19/08/2008	02/08/2008 08:08	11/08/2008 11:45	06/09/2008 17:04	35.4	300.9	270.4	1.8	3.7	0.6
17	16/12/2009	03/12/2009 00:28	17/12/2009 15:20	07/01/2010 16:41	35.7	493.1	452.3	5.8	8.2	1.9
18	16/02/2010	26/01/2010 18:46	09/02/2010 14:40	27/02/2010 01:27	31.3	54.0	19.1	2.3	2.7	0.9
19	31/05/2010	10/05/2010 19:28	29/05/2010 15:50	15/06/2010 23:40	36.2	109.0	72.8	2.6	2.5	0.8
20	13/04/2011	31/03/2011 14:07	15/04/2011 10:50	02/05/2011 23:11	32.4	320.8	288.6	2.3	4.1	0.8
21	08/03/2012	14/02/2012 06:58	08/03/2012 15:40	21/03/2012 13:33	36.3	328.2	287.3	7.3	34.2	2.3
22	01/09/2012	24/08/2012 16:26	04/09/2012 15:15	15/09/2012 10:18	21.7	180.0	156.1	0.8	1.0	0.4
23	15/12/2016	03/12/2016 04:34	12/12/2016 16:05	28/12/2016 01:00	24.9	1560.0	1539.8	1.9	3.0	0.9
24	09/03/2017	11/02/2017 10:06	09/03/2017 16:10	30/03/2017 06:56	46.9	87.0	45.4	4.1	5.8	1.0
25	16/06/2017	09/06/2017 17:46	26/06/2017 15:40	12/09/2017 23:24	95.2	109.0	71.5	4.8	1.9	0.6
26	20/12/2017	21/12/2017 19:22	22/12/2017 02:45	07/01/2018 04:41	16.4	178.5	99.8	0.4	0.4	0.3
27	20/01/2019	24/01/2019 08:01	26/01/2019 16:10	30/01/2019 22:18	6.6	400.6	382.1	0.2	2.7	0.3
28	15/03/2019	06/03/2019 19:19	15/03/2019 10:30	27/03/2019 18:59	21.0	47.8	34.9	0.9	0.8	0.5
29	N.d	05/12/2019 22:01	12/12/2019 10:30	24/12/2019 02:32	18.2	272.0	253.1	0.6	0.4	0.4

Volumes, TADR and MOR are calculated as bulk values (see “Methods”).

*Date of explosion from Ozerov et al.^41^.

**Event #15 not detected by MODIS but reported in Ozerov et al.^41^.

### Klyuchevskoy

**Figure 3:**
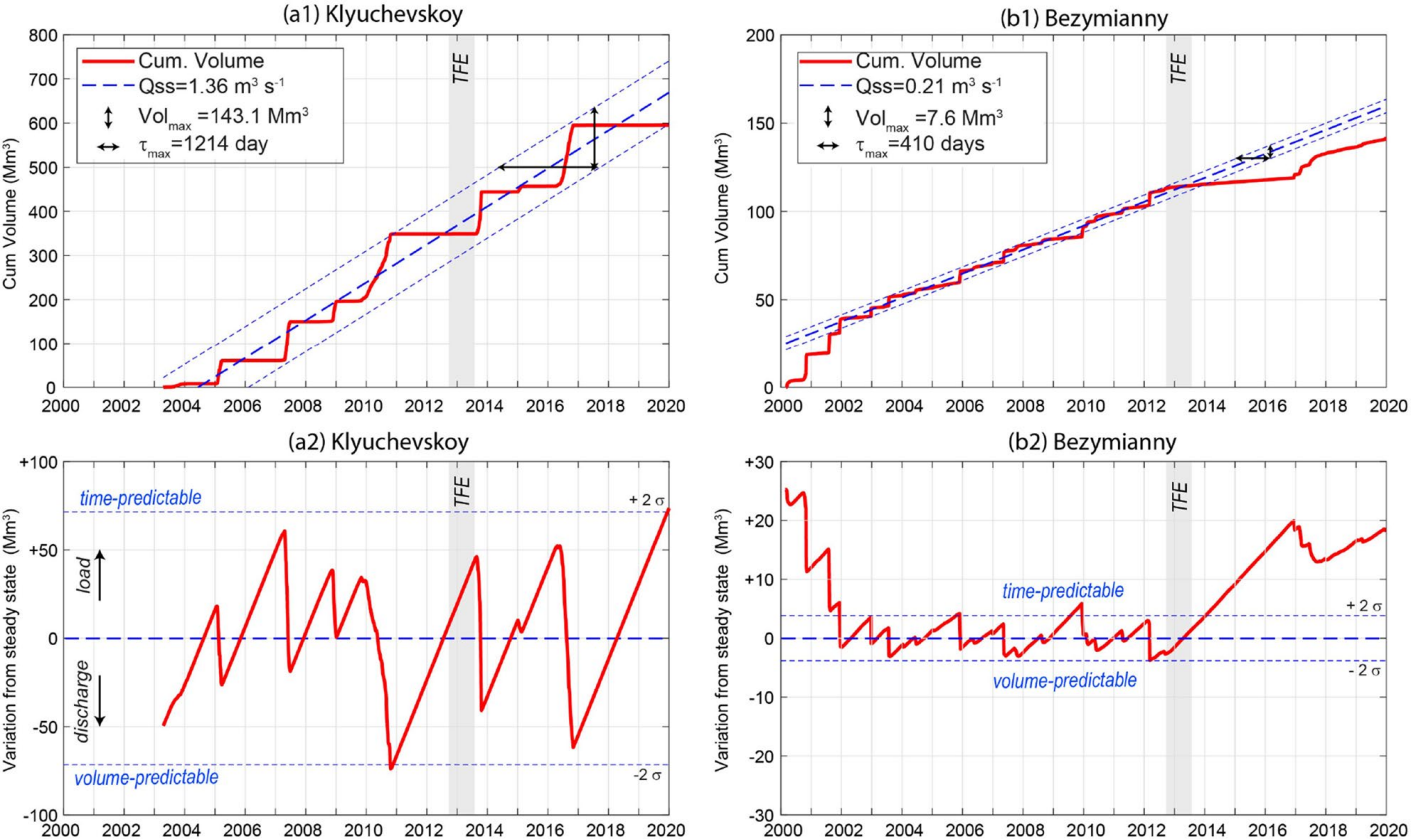
*Upper panels.* Cumulative volume curves for (**a1**) Klyuchevskoy and (**b1**) Bezymianny, derived from satellite measurements (see “Methods”). The linear fits of each cumulative curve represent the linear growth model (blue thick dashed lines) and provide the steady-state output rate (Q_ss_). The two parallel lines (linear model ± 2σ) define the maximum eruptible volume (Vol_max_) and the maximum response time (τ_max_) for the inferred steady-state condition. *Lower panels*. Load and discharge model for (**a2**) Klyuchevskoy and (**b2**) Bezymianny. The variation from the steady-state is calculated as the residual between the linear growth model and the observed cumulative volume curves. A two-fold standard deviation (± 2σ) of residual is used to define the upper and lower volume limits for the load-discharge model. In this plot, a time-predictable system would have a near-constant upper threshold of the volume load when eruptions occur. A volume-predictable system would have a near-constant lower threshold. An entirely predictable system would have both. The gray bar indicates the timing of the 2012 Tolbachik Fissure Eruption (TFE). Time series generated using MATLAB software (www.mathworks.com).

**Figure 4:**
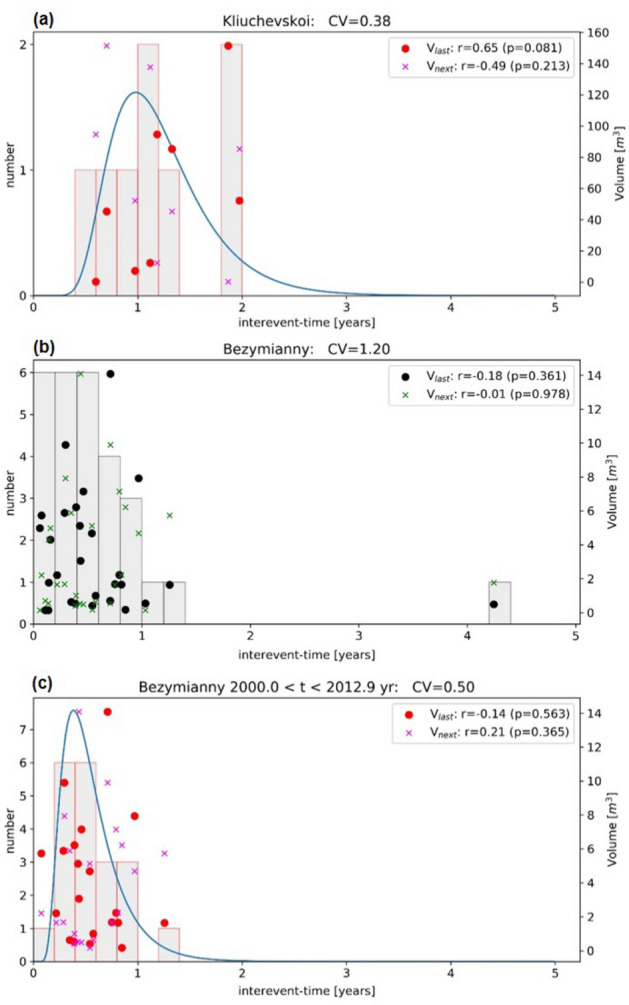
Inter-eruption times distribution (bars) for (**a**) Klyuchevskoy, (**b**) Bezymianny—whole period (2000–2020), (**c**) Bezymianny—pre-Tolbachik eruption (2000–2012). The coefficient of variation (CV), a measure for the eruption’s periodicity, is provided in the title of each plot. Symbols show the eruption volume (right axis) in all three panels as a function of the inter-eruption time, where points and crosses refer to the volume of the last and next event, respectively. The correlation coefficient (r) between volume and interevent-time with its *p *value is provided in the legends.

Nine eruptions occurred at Klyuchevskoy between 2003 and 2020 (Fig. 2a). Of these, eight were automatically recognized (see “Methods”), and one was manually selected, based on observations of Ozerov et al.^41^ (eruption #6; Tables 1, 2). Most of the eruptions (#2, 3, 4, 5, 7, 8, 9; Table 1) produced lava flows along the flanks of the volcano^41^ and created lava volumes ranging from ~ 10 to 150 × 10^6^ m^3^ each, with a mean output rate (MOR: total volume of eruption/duration) ranging between 2.5 and 10 m^3^ s^−1^ and a maximum TADR often higher than 30 m^3^ s^−1^ (Table 1). Only two eruptions (#1, 6; Table 1) were limited to moderate explosive activity inside the summit crater^41^ characterized by much lower volumetric output (< 10 M m^3^) and discharge rates (maximum TADR < 2.5 m^3^ s^−1^; Table 1). For some eruptions (#3, 4, 5; Fig. 2a), the onset of lava effusion was preceded by a precursory phase of several weeks, identified by increased fumarolic activity and degassing^41^. In other cases, the beginning of the eruption was rather rapid, without any apparent thermal precursory phase (#2, 7, 8, 9; Fig. 2a). The eruptive trends of Klyuchevskoy are often characterized by a TADR that increases with time to reach values of 10–100 m^3^ s^−1^ immediately before the effusion suddenly ceases (Fig. 2a). The volumetric output of the 20 years (Fig. 2) defines the most recent period of intense activity of Klyuchevskoy characterized by a steady-state output rate (Q_ss_) of 1.36 m^3^ s^−1^ (1.21 m^3^ s^−1^ DRE; Fig 3a), which is almost twice the average output since 1930 (Fig. 1b1). Notably, the cumulative curve in Fig. 3a shows a clear sawtooth pattern typical for steady-state volcanism^61^ whereby each step is either produced by (i) an unbuffered arrival and eruption of discrete magma batches, or (ii) a partial or complete discharge of a shallow reservoir that is fed by a constant magma supply. A similar pattern could be also explained if the arrival of discrete magma batches is controlled by a steady-state destabilization of magma reservoirs, produced by passive degassing during quiescence, which can trigger magma ascent from depth^62–64^. Whatever the model, the two lines, which envelope the sawtooth curve (parallel to the linear trend ± 2σ; Fig. 3a), define the maximum size (maximum eruptible lava volume) and maximum response time of the individual eruption, respectively^61^. For Klyuchevskoy, these values are approximately 143 × 10^6^ m^3^ and 1214 days. The analysis of inter-eruption time distribution (“Methods”) suggests a relatively strong periodicity (Fig. 4a). The degree of periodicity can be quantified by the coefficient of variation (CV), which is zero for perfect periodicity, one for randomness, and larger than one for clustering. In the case of Klyuchevskoy’s eruptions, we found a CV equal to 0.38 and the inter-eruption time positively correlated to the size of the last event (correlation coefficient of 0.65), as expected for time-predictable systems (Fig. 4a). A load and discharge model is thus envisaged for Klyuchevskoy (Fig. 3), whereby an eruption starts when the upper, critical volume threshold is accumulated in the shallow reservoir^65^. The resumption of eruptive activity on November 2019^66^ further supports a time-predictable behavior (“Methods”), which is in agreement with the achievement of a critical volume as shown in Fig. 3a.

### Bezymianny

Thermal data acquired over Bezymianny (Fig. 2b) are indicative of an open-vent volcano, persistently emitting hot volcanic products. The retrieved long-term eruptive pattern can be subdivided into two distinct regimes: (1) a continuous low-level regime, associated with passive degassing and possibly related to “endogenous growth,” and (2) an intermittent high-level regime, associated with short-term (days to weeks) extrusive-explosive-effusive cycles. A TADR threshold of 0.25 m^3^ s^−1^ separates the two regimes and automatically recognizes 28 out of the 29 major eruptive cycles between 2000 and 2019^41,44^ (Table 2). The only undetected event occurred on 5 November 2007, when strong cloud coverage over the volcano prevented the detection of this short-lived event (Tables 1, 2). Each eruption cycle is characterized by erupted volumes ranging from ~ 0.15 to ~ 15 × 10^6^ m^3^ and peak TADRs between 0.35 and 52 m^3^ s^−1^ (Table 2). The average duration of each eruptive cycle is 26.7 (± 20.7; 1σ) days, much shorter than the average inter-eruption time of 222.7 days. The cumulative volume curve of Bezymianny is essentially controlled by the sudden steps associated with the eruptive cycles detected by MODIS (Fig. 3c). Between 2000 and mid-2002, eruptions reached higher TADR peaks, causing a steeper cumulative volume curve than in the rest of the time series (Fig. 3c). Although this may reflect a higher magma output rate in this period, it is also possible that the dataset is biased by the fact that only one satellite was operating in that period (see “Methods”). Similar to Klyuchevskoy, the cumulative curve of the volumes erupted between May 2002 and September 2012 suggests steady-state volcanism for Bezymianny^61^, which is characterized by an average output rate of ~ 0.21 m^3^ s^−1^, a maximum eruptible volume of ~ 7.6 × 10^6^ m^3^, and a maximum repose time of 410 days (Fig. 3c, d). A notable lack of eruptive cycles occurred between September 2012 and December 2016 (Fig. 2b). This anomalously long rest period (low thermal regime) is also visible in Fig. 3b, c, where the cumulative volume curve diverges horizontally from the steady-state model. According to Wadge^61^, this pattern occurs at steady-state volcanoes when magma is not being supplied into the shallow reservoir, here either because magma is not being generated or a neighboring volcano is capturing it. Bezymianny's activity resumed at the end of 2016 and continued intermittently with an output rate similar to the 2002–2012 period. The analysis of inter-eruption times (“Methods”) suggests a quasi-periodic behavior (CV = 0.5) for Bezymianny’s activity until 2012 (Fig. 4b), which is completely lost when including the whole dataset (Fig. 4c). No correlation is found between the inter-eruption times and volumes released during the last or the next eruption (Fig. 4). Although the lack of correlation can be due to the significant uncertainties affecting the Bezymianny time series (“Methods”), we may not exclude the role of a time-varying upper threshold (strength) of the shallow magmatic system^65^.

### Tolbachik

The eruption of Tolbachik (November 2012–August 2013) commenced suddenly on 27 November 2012, producing an initial TADR peak of about 300 m^3^ s^−1^. This initial activity resulted in the emplacement of a lava flow that reached a length of about 15 km in a few days^67^. Effusion rates decrease roughly exponentially during the following ten months of continuous effusive activity. The eruption stopped between 23 and 27 August 2013 when the TADR suddenly lowered from 7 to 9 to less than 0.25 m^3^ s^−1^. The eruption emplaced a volume of ~ 0.5 × 10^6^ m^3^ in 302 days^58^, which is up to 4000 times the volume commonly erupted at Bezymianny volcano (> 0.15 × 10^6^ m^3^, see above).

## Interactions between Klyuchevskoy, Bezymianny, and Tolbachik

We statistically explore if the three volcanoes interacted on more than one occasion and in different ways. Specifically, we found various degrees of interactions that become best observable by analyzing the data at the time scales from weeks to decades.

Below we provide evidence of interactions related to (i) conjoint activity of Klyuchevskoy and Bezymianny throughout 2003–2020, (ii) the reactivation of Tolbachik in 2011–2012, (iii) the reactivation of Klyuchevskoy and the cessation of Tolbachik in August 2013, (iv) the reactivation of Bezymianny in 2016–2017, and (v) changes in the long-term magma output rate after the Bezymianny eruption, in 1955–56, and after the Great Tolbachik Fissure Eruption (GFTE), in 1977.

### Conjoint activity and pattern’s change before and after the 2012 Tolbachik eruption

**Figure 5:**
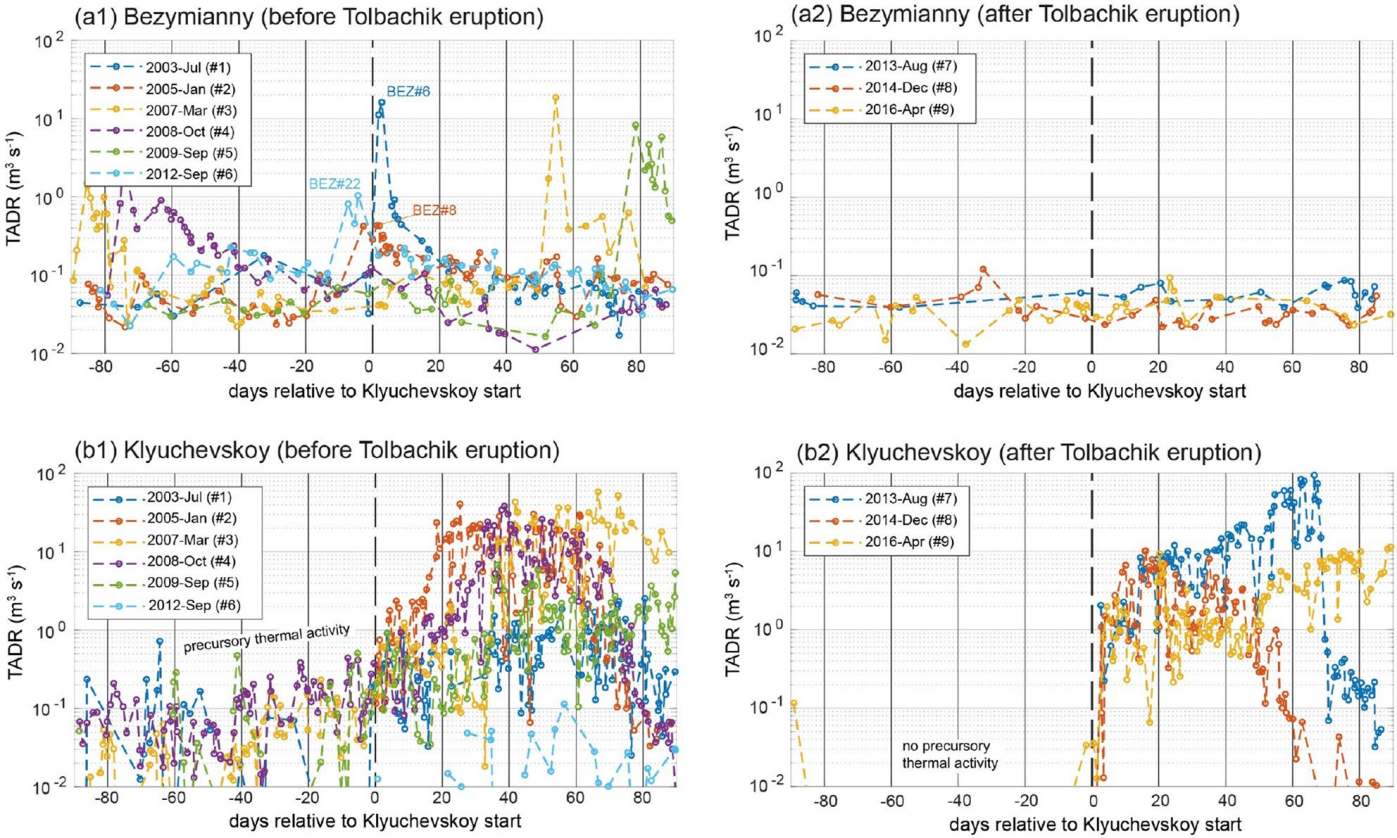
Stacked TADR time-series relative to the onset of the Klyuchevskoy eruptions, before (left column) and after (right column) the Tolbachik eruption. *Upper panels*: TADRs of the Bezymianny volcano. *Lower panels*: TADRs of the Klyuchevskoy volcano. The simultaneous activation of Bezymianny at the time of the eruption onset of Klyuchevskoy is obvious before the Tolbachik eruption (**a1**) but absent afterwards (**a2**). Similarly, at Klyuchevskoy, the precursory activity is evident for the eruptions that occurred before the Tolbachik eruptions (**b1**), but not afterward (**b2**). The time window of ± 90 days is chosen for visualization purposes, because most of the Klyuchevskoy eruptions show their maximum within this time after the eruption onset. Time series generated using MATLAB software (www.mathworks.com).

A first indication of how volcanoes are interconnected with each other is revealed by the detailed analysis of the mutual activity of Klyuchevskoy and Bezymianny (and pattern’s change) before and after the Tolbachik eruption (Fig. 5).

Before the latter (Fig. 5a1), we observe a simultaneous activation of Bezymianny and Klyuchevskoy several times (i.e., eruptions KLY#1, 2, 6) while no simultaneous activation is found afterward (Fig. 5a2). In particular, the onset of Klyuchevskoy's eruptions #1, 2, 6 coincided with the maximum activity of Bezymianny (BEZ#6, 8, 22) in the same period (a time window of ± 10 days is considered to avoid the effect of clouds). Although less significant, Bezymianny's activity also showed some synchronous activation (increase of TADR relative to the previous trend) with the onset of the other Klyuchevskoy eruptions (KLI#3, 4) before the Tolbachick eruption.

More specifically, we found that in the days-to-weeks following each of Klyuchevskoy eruptions, the average TADR of Bezymianny increased, on average, by a factor of four (“Methods”). This increase suggests that before 2012, the eruptions of Klyuchevskoy were able to “galvanize” also the activity of Bezymianny. In contrast, after the eruption of Tolbachik, the two volcanoes no longer have erupted simultaneously.

Additionally, before the Tolbachik eruption, most of the eruptions of Klyuchevskoy were characterized by a precursory phase marked by a gradual increase in thermal activity and estimated TADR (Fig. 5b1). This pre-eruptive pattern is typical of open-vent volcanoes, in which the rise of the magma column causes the appearance and growth of fumaroles or weak explosive activity^68^. However, the precursory pattern disappeared after the eruption of Tolbachik (Fig. 5b2), and all the three subsequent eruptions of Klyuchevskoy showed a sudden beginning of activity more typical of closed-vent systems^68^.

### Reactivation of Tolbachik in 2011–2012

**Figure 6:**
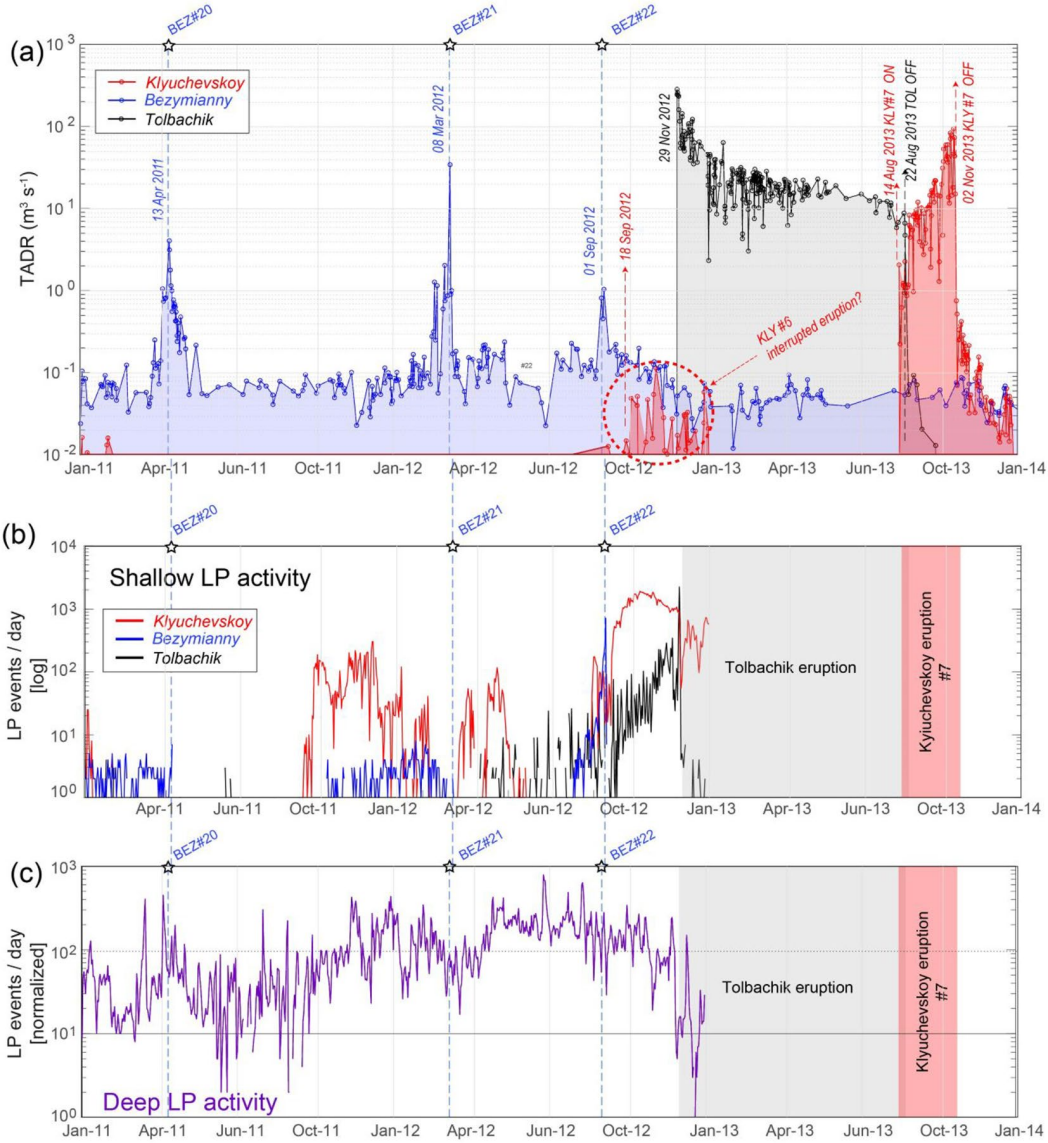
Reactivation of Tolbachik and responses at other volcanoes. (**a**) Stacked TADR time-series for Klyuchevskoy, Bezymianny, and Tolbachik between 2011 and 2014. (**b**) Shallow LP seismicity (normalized number of events per day) below the Klyuchevskoy, Bezymianny, and Tolbachik during the same period (modified from Shapiro et al. 2017); (**c**) Deep LP seismicity below the KVG (seismic data modified from Shapiro et al. 2017); (refer to the color version of the figure). Time series generated using MATLAB software (www.mathworks.com).

The comparison between the surface activity, retrieved from satellite, and the long-period (LP) earthquakes occurred within the KVG during the reactivation of Tolbachik^22,69^, provides further indications of mutual communication between these volcanoes. This last was preceded in 2011 by an increase of the deep long period (DLP) seismic activity reaching its maximum level in May 2012 (Fig. 6c). It reflected the gradual pressurization of the whole KVG plumbing system^69^, possibly in response to a pulse of volatile-rich basaltic magmas rising from the mantle^70^. At Bezymianny, this gradual pressurization may have triggered three consecutive shallow LP swarms, each preceding an eruption, the last one being in September 2012 (Fig. 6a, b). Similarly, LP seismicity also migrated shallow below the Klyuchevskoy volcano in September 2012 (promptly triggering the onset of eruption #6), and later, on October–November 2012, LPs occurred below Tolbachik, just before the onset of its voluminous flank eruption (Fig. 6a, b).

Interestingly, during eruption #6 of Klyuchevskoy, the TADR trend almost mirrors the shallow LP seismicity, both reaching a maximum in November 2012 and then declining in correspondence with an acceleration of the Tolbachik seismic swarm (Fig. 6a,b). Eruption #6 was somehow atypical for recent activity of this volcano since it produced only weak Strombolian activity^41^, with a TADR always below 0.25 m^3^ s^−1^ and a volume of less than 1 × 10^6^ m^3^ (Tables 1, 2). Moreover, unlike the other Klyuchevskoy eruptions (cf. Fig. 2a), it never culminated in effusive activity, which is atypical for this volcano. Together with a waning trend of surface and seismic activity since mid-November 2012, these peculiar features suggest a sort of partial depletion of the shallow magma supply of Klyuchevskoy, precisely in correspondence with the acceleration of seismic swarms below Tolbachik (Fig. 6a, b). It is worth noting that the eruptions of Klyuchevskoy stopped in 1975–1976 during the GFTE and were renewed in 1977–1978 after the GFTE^71^.

### Reactivation of Klyuchevskoy and cessation of Tolbachik eruption in August 2013

Even more intriguing is the resumption of the activity at Klyuchevskoy (eruption #7) and the almost concurrent cessation of activity at Tolbachik on 22 August 2013 (Fig. 6a). The beginning of eruption #7 occurred suddenly on 14 August 2013, with the onset of Strombolian explosions, which evolved in few days into summit effusive activity^41^ fed with a TADR of about 10 m^3^ s^−1^ (Fig. 6a). Lava discharge rates increased rapidly in the following months to reach a maximum value of ~ 100 m^3^ s^−1^ on 18 October 2013, just before the abrupt cessation of surface activity on 25 October 2013.

The onset of eruption #7, which also occurred abruptly on 14 August 2013, preceded the end of the Tolbachik eruption by eight days (Fig.6a). Our data suggest that the Tolbachik eruption ended when the TADR-values were still moderately high (7–9 m^3^ s^−1^), shutting down the monthly-long, (almost) exponential decay.

### Reactivation of Bezymianny in 2016

**Figure 7:**
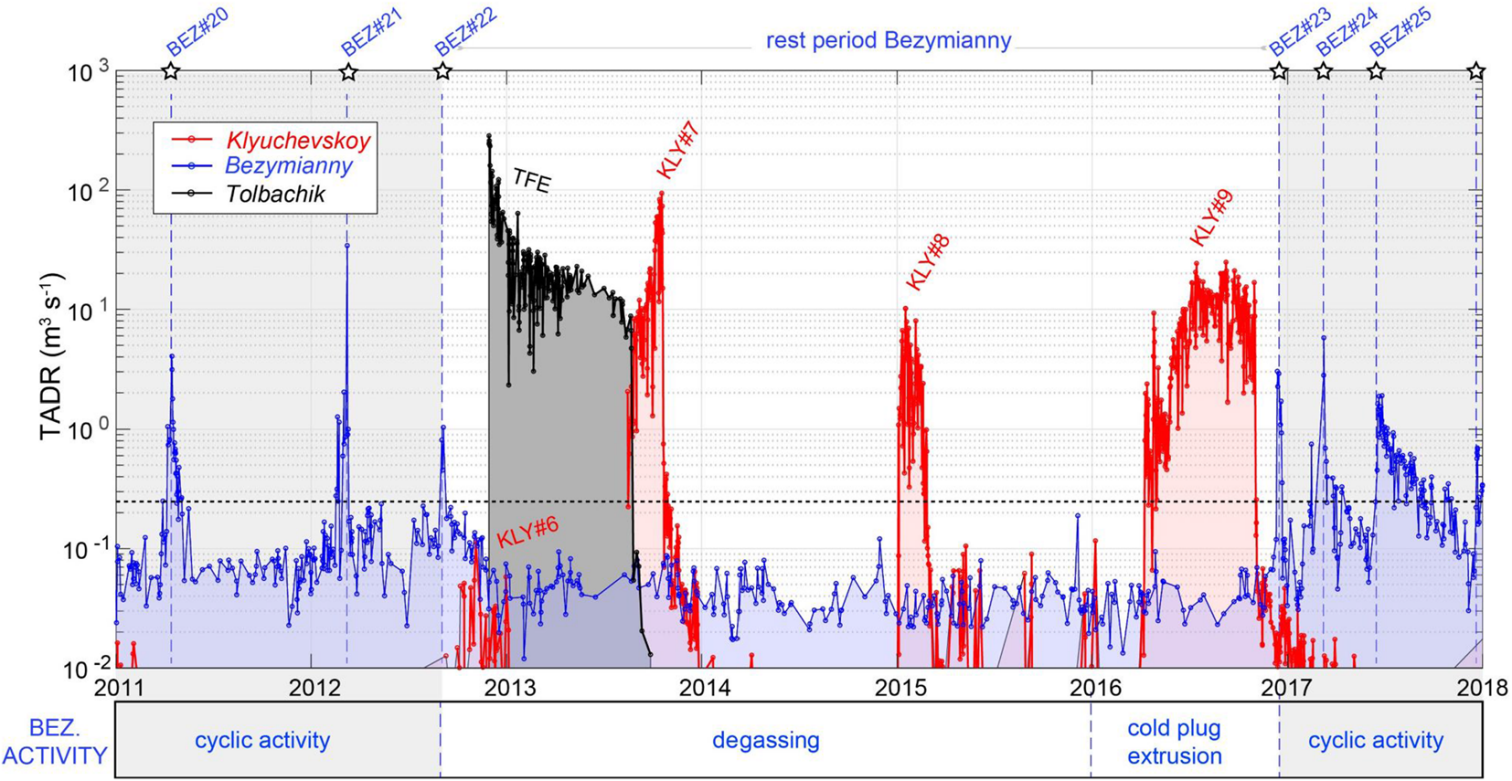
Stacked TADR time-series for Klyuchevskoy, Bezymianny, and Tolbachik volcanoes between 2011 and 2018. Eruptive periods for each volcano are labeled according to Tables 1 and 2. Gray fields outline periods of Bezymianny activity characterized by frequent explosive eruptions (stars); The reactivation of Bezymianny started in early 2016, through the extrusion of “cold” crystallized plug, undetected by MODIS. This anomalous precursory phase culminated in December 2016 (#BEZ23) with the effusion of a new lava flow that marks the resumption of activity of Bezymianny after the Tolbachik eruption. Time series generated using MATLAB software (www.mathworks.com).

Strong evidence for volcano-volcano interactions is the lack of the typical extrusive-explosive-effusive cycles of Bezymianny for four years after the eruption of Tolbachik^72^ (Fig. 7). This rest period was unusually long for Bezymianny (1550 days) and started already on 11 September 2012 (~ 3 months before Tolbachik). As discussed above, the September 2012 eruption of Bezymianny represents the superficial response of its plumbing system to the main deep magma pulse revealed by DVLP, which heralded a few months later the eruption of Tolbachik. The following lack of activity at Bezymianny persisted for four years during which continuous thermal anomalies were likely related to passive degassing (Fig. 7). In early 2016, a viscous, crystallized, cold plug started to be extruded from the summit crater^73^. This slow, cold extrusion was undetected by MODIS but, according to Mania et al.^73^, accelerated in September-November 2016 (right at the end of Klyuchevskoy eruption #9) until the effusion of a viscous lava flow on 9 December 2016 (eruption #23; Fig. 7). The extrusion of solid plugs at the onset of eruptive cycles is a typical feature of Bezymianny^41,44^. However, that of 2016 represented an abnormally long precursory phase for this volcano. It was followed by eruptions #24 and #25, both characterized by a gentle effusion of two lava flows with increasingly stronger explosivity^73^. This peculiar dynamic after four years of rest seems to be consistent with an interruption (or decrease) of the magma supply after the TFE that favored the formation of a cold crystallized plug in the shallow conduit of Bezymianny.

### Influence of the 1955–56 eruption of Bezymianny and the 1975 Great Tolbachik Fissure Eruption in the long term eruptive pattern of Klyuchevskoy and Bezymianny

Bezymianny eruption in 1955–56 (BEZ55) was the largest in the recent history on this volcano^43^ and its occurrence may have perturbed the whole KVG in some way. Some evidence appears from the analysis of the long term volumetric output of Klyuchevskoy (Fig. 1b) which shows an evident reduction in the eruption’ frequency after 1956, passing from 0.28 events/year, between 1930 and 1953, to 0.17 events/year between 1956 and 1973 (Fig. 1b). Even more indicative is the fact that this decrease was also associated with an evident geochemical change in the products erupted by Klyuchevskoy after 1960^42^ (a few years after the unrest of Bezymianny), which has been ascribed to the injection of new type of primary magma that was not produced beneath the volcano previously.

In 1977, immediately after the GTFE, the eruptive regime of Bezymianny changed considerably to give rise, for the first time, to the effusion of lava flows and the establishment of extrusive-explosive-effusive cycles^27,45^. Simultaneously, the volumetric output rate decelerated (Fig. 1c). The erupted magma became more and more primitive, indicating the arrival of deeper mafic magma components at the surface^54^. For Klyuchevskoy, the available data and observations point to a significant change in its eruptive regime starting 1–3 years after the GTFE, when summit eruptions began to dominate over flank eruptions^40^ (Fig. 1b). In contrast to Bezymianny, a significant increase of the output rate accompanied this change likely associated with an increased magma supply at shallower levels. Geodetic measurements^40^ carried out between 1979 and 2005 also suggest that the magma feeding system of Klyuchevskoy was accumulating considerable amounts of magma before the eruptions of this period, which is coherent with a gradual rise of the effective pressure source, from the probable region of deep magma storage (25 km) to shallow levels (5 km). Also the seismicity of the entire KVG showed a dramatic change after the GTFE eruption^22,71^ with several remarkable earthquake swarms that occurred during 1977–1978. All these pieces of evidence make it plausible that both the BEZ25 and GTFE have perturbed the activity of the neighboring volcanoes, compatibly with a process of general rejuvenation of the whole KVG magma system. Whether a similar rejuvenation process occurred during the 2012 Tolbachik eruption is still unclear. However, the occurrence of the major swarms of deep very-long period events (DVLP) in 2011 and 2012 (Fig. 6) point toward a process of pre-eruptive reload of the shallow magmatic reservoirs from depth^23^.

## Discussion

Our new satellite data suggest that the three volcanoes of the KVG are related to each other on various time-scales. The mode and directivity of the relation vary, showing correlated and anti-correlated activity changes. This observation probably reflects a complex response to changes occurring in a seismically inferred common magmatic source and/or at the associated hydrothermal system.

Conjecturing the presence of crustal magma chambers at the volcano systems, we may develop a simple conceptual model to explain some of the modulations and concurrent activity changes observed in our data. At Klyuchevskoy, the magma supply within the crustal plumbing system follows a general steady-state load and discharge model. The frequent but intermittent arrival of magma batches is buffered by the elastic deformation of the subvolcanic reservoir^61^. Eruptions occur when the stored amount of magma exceeds a specific threshold (time-predictable behaviour^65^) with the maximum eruptible volume (~ 150 × 10^6^ m^3^ for Klyuchevskoy; Fig. 3a2) strictly connected to the capacity of the reservoir to buffer the arrival of magma^54^. It is interesting to note that during the steady-state regime, the magma ascent feeding the activity at these volcanoes could be driven by processes occurring at depth^61^ (down-top mechanism), but also by the passive degassing during quiescence^62–64^, which induces the opening of pathways connecting deep and shallow magma reservoir (top-down-mechanism).

The eruptive behavior of Bezymianny is also compatible with a steady-state magma supply. However, in this case, the smaller capacity of the reservoir(s) and the lower magma supply rate (compared to Klyuchevskoy) give rise to much more frequent but less voluminous eruptions (maximum eruptible volume ~ 7 × 10^6^ m^3^; Fig. 3b2). In this steady-state framework, the volcanoes’ conjoint activation indicates that both systems responded to a common perturbation, possibly sourced at lower crustal levels.

On the other hand, significant large swarms of DVLP (Fig. 6), ascribed to deep magma pulses, can reactivate the Tolbachik magmatic path^22,69–71^, which in turn modify the properties of the of nearby magmatic systems and perturb their steady-state regime.

Deviation from the steady-state cumulative volume curve indicates a change in the magma supply rate^61^, as occurred after the BEZ55 and the GTFE at both Klyuchevskoy and Bezymianny.

The GTFE eruption directly affected Bezymianny’s activity, causing a reduction of the magma output rate since 1977 (Fig. 1b2) and producing a radical change in Bezymianny’s eruptive regime and a rejuvenation of its eruptive products^54^. Similarly, but in the opposite direction, the GTFE led to an increase of the long-term magma output rate of Klyuchevskoy and promoted a change in its eruptive pattern, switching from lateral to summit eruptions (Fig. 1b1).

To a lesser extent, the reactivation of the Tolbachik in 2012 inhibited the steady-state magma supply of Bezymianny for several years. It caused the interruption of its surface activity until the extrusion of a crystallized plug in 2016 (Fig.7). During this period, multiple interactions between Tolbachik and Klyuchevskoy were also observed, supporting the existence of a very efficient connection between the plumbing systems of the three volcanoes. We note that the details on the presence of a common primary magma feeding all volcanoes in KVG as well as the location and geometry of crustal magma chambers are still debated^19,39^, which is why our conceptual model remains speculative.

Shapiro et al.^22^ proposed a model based on fluid-pressure propagation through porous rocks to explain the migration of LP events and infer the existence of such hydraulic connections below the KVG volcanoes. Our data supports and reinforces this hypothesis, although we may not exclude that elastic stress changes in the crust, controlled by the eruptions, would also explain the connection among volcanoes and their dynamics^74^. Independent of any model assumption, our data show that the magmatic systems below the KVG are interconnected, and eruptions of individual volcanoes can be the direct consequence of their neighbors’ activity.

To what extend magmatic systems are connected and if one eruption can trigger another volcano are essential questions for assessing volcanic hazard. In the case of interacting volcanoes, such as in the case of KVG, a volcano’s behavior can be the direct consequence of its neighbor’s activity. In these cases, traditional hazard assessments of isolated volcanoes have to be replaced by a comprehensive assessment involving the whole volcanic group. In addition to its eruptive history, the volcano's hazard assessment has to account for its neighboring volcanoes' eruptive history, which may influence its current state.

## Methods

### Satellite thermal data

Satellite thermal data were processed using the MIROVA system^60^ (www.mirovaweb.it), which is based on the analysis of the images acquired by MODIS. The two MODIS sensors, launched in March 2000 and May 2002, provide approximately six infrared images per day over Kamchatka (three night-times and three day-times) with a nominal ground resolution of 1 km. MODIS images are processed at each volcano to quantify the Volcanic Radiative Power (VRP in Watts), a combined measurement of the area and integrated temperature of the hot (> 200 °C) volcanic features with a standard error of ± 30% over every measurement^60^.

We used only the night-time MODIS dataset, consisting of approximately 19,500 images acquired over the Klyuchevskoy Volcanic Group (KVG). Thermal anomalies detected by MIROVA were geolocated (errors in geolocation are less than 0.5 km for nadir acquisition^60^) to discriminate the hotspots sourced by the three distinct volcanoes. All the images were visually analyzed to discard the data contaminated by clouds, ash plumes, or poor viewing conditions (i.e., high satellite zenith), which preclude a correct estimation of VRP^15,53^. Finally, the supervised dataset consists of 2139 images for Klyuchevskoy, 2013 images for Bezymianny, and 219 images for Tolbachik, which have been used to reconstruct the time-series of VRP (Fig. S1—Supplementary Material). For each volcano, the cumulative Volcanic Radiative Energy (VRE) in Joules is calculated as the trapezoidal integration of the supervised VRP time series (Fig. S1—Supplementary Material).

### Erupted volume and time-averaged lava discharge rate

We used a simplified approach, which has been expressly developed to derive time averaged lava discharge rate (TADR) directly from MODIS-derived VRP^75^. This approach assumes that during an eruption, the energy radiated by a lava body (i.e., VRE) is linearly correlated to the bulk erupted volume (Vol),

1 $$c_{{rad}} = \frac{{VRE}}{{Vol}},$$

where c_rad_ (in J m^−3^) is the best-fit coefficient that describes the ability to radiate thermal energy by unit volume of the observed lava body. Thus the c_rad_ value can be determined retrospectively by measuring the energy radiated during an eruption (or during an eruptive period) and the bulk volume of the lava flow(s) or domes emplaced during the same time interval (measured independently).

Once calibrated, the c_rad_ coefficient is used to retrieve the TADR for each single VRP measurements according to

2 $$TADR = \frac{{VRP}}{{c_{{rad}} }}.$$

Note that this approach does not take into account the volume of magma erupted explosively (i.e., ash plumes, pyroclastic density currents). It accounts only for magma erupted during effusive/extrusive periods, that is, when sufficient thermal radiation is detectable from the satellite.

To estimate the c_rad_-value of Klyuchevskoy, we considered the period between 2002 and 2009, during which about 231 × 10^6^ m^3^ of lava erupted^21^. Assuming an average fraction of tephra equal to 15% in volume^40^, the cumulative volume of lava flows erupted between 2002 and 2009 become ~ 196 × 10^6^ m^3^. This activity produced a VRE of 1.6 × 10^16^ J (Fig. S1c1—Supplementary Material), which results into an average c_rad_-value of 8.16 × 10^7^ J m^−3^.

For Bezymianny volcano, we calibrated the c_rad_, by considering the dome volume’s growth between 31 July 2006 and 9 September 2017^27^. Given a total volume of ~ 69 × 10^6^ m^3^ and a VRE of 1.17 × 10^15^ J (Fig. S1c2—Supplementary Material), we estimated c_rad_ = 1.88 × 10^7^ J m^−3^. Note that the TADR and inferred volumes do not include the contribution of the explosive activity, which in the case of Bezymianny may be relevant. According to Girina et al.^44^, each extrusive-explosive-effusive cycle produces volumes up to ~ 10^7^ m^3^, in the form of pyroclastic flows. Although the amount of juvenile material inside these deposits is unknown, the large amount of material erupted explosively, together with the short duration of each cycle, introduces a significant level of noise into our time series and an uncertainty possibly higher than 100% in the volumes reported in Table 2.

Equations (1) and (2) have been successfully applied to estimate the TADRs of the 2012–2013 Tolbachik eruption^67^, where a c_rad_ equal to 1.08 × 10^8^ J m^−3^ has been calculated based on a final lava flow volume^76^ of 573 × 10^6^ m^3^ and a corresponding VRE equal to 6.07 × 10^16^ (Fig. S1c2—Supplementary Material).

As described by Coppola et al.^75^, this approach provides single TADR measurements with an associated error of ± 50%. Errorbars are not shown for graphical convenience.

### Statistical testing of correlated activity

The frequency plot of inter-eruption time (dt_es in Tab 1) for Bezymianny and Klyuchevskoy is shown in the left axis of Fig. 4. The peaked distribution for Bezymianny data (gray bars in Fig. 4a, b) can be reasonably fitted by a Brownian-passage time (BPT) distribution (blue line). This models assumes a fixed eruption threshold and volume release, plus a constant loading rate with noise. The coefficient of variation (CV), also called the aperiodicity parameter, measures a signal’s periodicity, where CV = 0 refers to a perfect periodicity, CV = 1 to a random Poisson occurrence, and CV > 1 to clustering. When considering the whole dataset of Bezymianny (Fig. 4a), the CV value is 1.20, indicating a random occurrence of eruptions. However, the CV value decreases to 0.50 when post-Tolbachik eruption data are excluded (Fig. 4b), thus indicating a quasi-periodic behavior until the Tolbachik eruption. For Klyuchevskoy (Fig. 4c), the CV value is even lower (CV = 0.38), indicating a rather clock-wise recurrence of eruptions.

The relation between inter-eruption time and volume release of the last or next event is shown on the right axis of Fig. 4. The data shows no correlations for Bezymianny (*p *values > 0.25 in Fig. 4a, b), while *p* < 0.05 would indicate a statistically significant correlation. In contrast, Klyuchevskoy (Fig. 4c) shows a positive correlation between the inter-eruption time and the volume of the last eruption (r = 0.65, *p* = 0.081), which become statistically significant (r = 0.73; *p* = 0.025) when including the timing of the last eruption (not included in our study) started in November 2019^66^. The weaker correlation with the next events’ volume found for the Kluchevskoi volcano (Fig. 4c) suggests that its eruption periodicity is consistent with a time-predictable rather than a volume-predictable model.

### Conjoint activity of Klyuchevskoy and Bezymianny volcanoes

**Figure 8:**
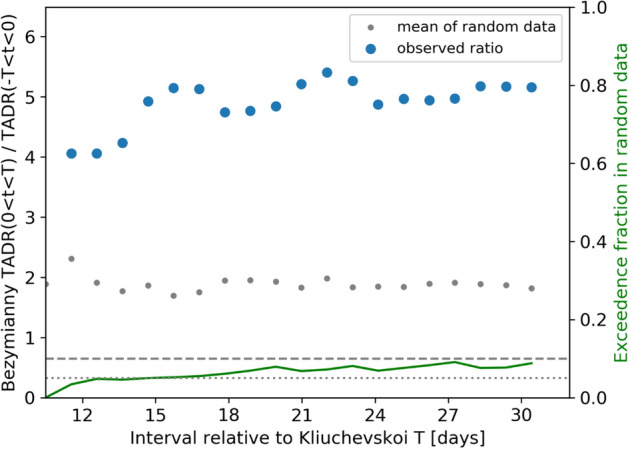
(Left Axis) Ratio between the averaged Bezymianny TADR values within T days after and before the Klyuchevskoy eruptions (#1–6). (Right axis). Mean fraction in randomized data that exceed the observed ratio (see the text for more explanations).

We tested the hypothesis that before the Tolbachik eruption, the activation of Klyuchevskoy (eruptions #1 to 6) affected the Bezymianny activity (Fig. S4). To perform this test, we first calculated the average TADR-value (of Bezymianny) within T days (from 10 to 30 days) relative to each Klyuchevskoy eruption and averaged those six values. Then we calculated the ratio between the averaged TADR-value in the T days after the eruption and the corresponding value in the T days before the eruption to measure the average activation (blue points in Fig. 8). Finally, we compared the observed ratio (as a function of T) with the corresponding result obtained after randomizing the activation times of the six Klyuchevskoy eruptions within the period between 2002 and the Tolbachik eruption. The fraction of randomized data with a ratio similar or larger than the observed one (green line in Fig. 8) shows that the observed activation value can only be reached in less than 5-10% of the randomized data. Although the results are close to the significance threshold, these data suggest that the result is significant for the shortest time intervals (i.e., T = 10 days; *p* < 0.05) with a 4–5 times increase of the averaged TADR of Bezymianny after the onset of a Klyuchevskoy eruption.

Note that we have not analyzed *T* < 10 days because of missing Bezymianny measurements during short periods before/after the Klyuchevskoy eruptions.

## Supplementary Information

Supplementary Information 1.

Supplementary Information 2.

## Data Availability

The satellite datasets are available as Supplementary Material.
