# Author Correction: Thermal remote sensing reveals communication between volcanoes of the Klyuchevskoy Volcanic Group

**DOI:** 10.1038/s41598-021-98440-8

**Published:** 2021-09-17

**Authors:** Diego Coppola, Marco Laiolo, Francesco Massimetti, Sebastian Hainzl, Alina V. Shevchenko, René Mania, Nikolai M. Shapiro, Thomas R. Walter

**Affiliations:** 1 Dipartimento Di Scienze Della Terra, Università Di Torino, Turin, Italy; 2 Centro Interdipartimentale Sui Rischi Naturali in Ambiente Montano E Collinare, Università Di Torino, Turin, Italy; 3 GFZ German Research Centre for Geosciences, Telegrafenberg, 14473 Potsdam, Germany; 4 Institute of Volcanology and Seismology FEB RAS, Piip boulevard 9, Petropavlovsk-Kamchatsky, 683006 Russia; 5 Institut de Sciences de La Terre, CNRS (UMR5275), Université Grenoble Alpes, Grenoble, France; 6 Schmidt Institute of Physics of the Earth, Russian Academy of Sciences, Moscow, Russia

Correction to: *Scientific Reports* https://doi.org/10.1038/s41598-021-92542-z, published online 22 June 2021

The original version of this Article contained an error in the spelling of the author Marco Laiolo which was incorrectly given as Laiolo Marco. The original Article and accompanying Supplementary Information files have been corrected.

