# Supplementary material for: Thermal remote sensing reveals communication between volcanoes of the Klyuchevskoy Volcanic Group
Source: Sci Rep. 2021 Jun 22;11:13090. doi: 10.1038/s41598-021-92542-z (PMC8219805; doi:10.1038/s41598-021-92542-z)
Supplement: Supplementary file 1 — Supplementary Information 1. [file 41598_2021_92542_MOESM1_ESM.pdf]

Supplementary material for the paper "The Kamchatka volcano orchestra – Remote sensing reveals communication between volcanoes of the Klyuchevskoy Volcanic Group "

Authors: Diego Coppola[1,2], Marco Laiolo[1,2], Francesco Massimetti[1,3], Sebastian Hainzl[3], Alina V. Shevchenko[3,4], Rene Mania[3], Nikolai M., Shapiro[5,6], Thomas R. Walter[3]

In this supplementary file, we present some technical details that support the conclusions presented in the main paper.

S.1 - Volcanic Radiative Power (VRP) and Energy (VRE) time-series

For each individual volcano, the data of Volcanic Radiative Power (VRP), Volcanic Radiative Energy (VRE), Time Average lava Discharge Rate (TADR) and cumulative erupted lava volume (Vol) are available in the Supplementrary TableS1.xls. A summary of VRP and VRE time series is provided in Fig. S1, where the periods used for the calibration of the card values (Methods) are reported.

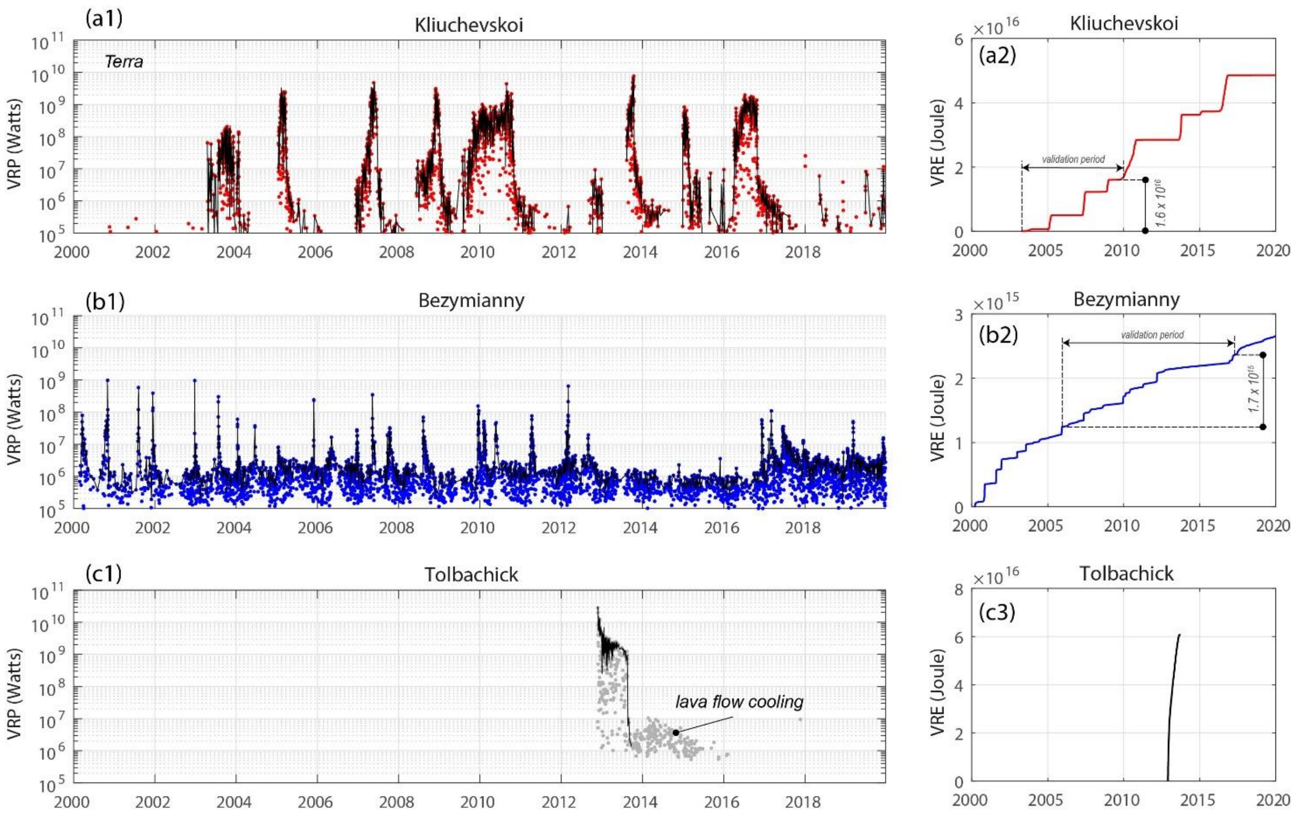

Figure S1. (#1 – left panels) Time-series of Volcanic Radiative Power (VRP) for (a) Klyuchevskoy, (b) Bezymianny, and (c) Tolbachik. Dots represent single measurements; Lines interpolate only supervised data (excluding cloud and poor-viewing geometry data). The two years-long tail after the 2012-2013 Tolbachik eruption is related to lava flow cooling. (#2 – right panels) Cumulative Volcanic Radiative Energy (VRE)

obtained by trapezoidal integration of supervised VRP time-series. The energy released during the validation period is used in combination with reported volumes to calculate the value of c_{rad} (see *Methods*).

S.2 - Automatic detection of eruptions

According to the following procedure, all the parameters reported in Table 1 were automatically extracted from the TADR time-series. First, we linearly interpolated the observed TADR values on an equidistant time grid with a spacing of 0.1 days. Then we used a seven-day long moving averaged filter with which the time-series has been convoluted (black lines in Fig. 2 and Fig. S1). Then, we applied a threshold equal to 0.25 $m^3 s^{-1}$ on the interpolated and smoothed time-series to identify the eruptive periods. An eruptive period is only considered as such if the convoluted TADR time-series remains above the threshold for at least five days to avoid spurious events. On the contrary, to avoid that long periods of bad weather cause the subdivision of a single eruption into several events, we grouped eruptive periods, which were separated by time intervals of less than 15 days (for Bezymianny) and 60 days (for Klyuchevskoy), into a unique eruption.

The peak time (peak in Table 1) is determined by the true maximum TADR (*TADRmax* in Table 1) value recorded in each eruptive episode, and the volume is the integrated volume rate of linear interpolated observation points. The Mean Output Rate (*MOR* in Table 1) is determined as the total volume erupted during a single eruption divided by its duration. Finally, inter-eruption times (*dt_es* in Table 1) are calculated as the time between the end of an eruption and the beginning of the following eruption.
